# Clinical exploration of the international society of limb salvage classification of endoprosthetic failure using Henderson in the application of 3D-printed pelvic tumor prostheses

**DOI:** 10.3389/fonc.2023.1271077

**Published:** 2023-12-14

**Authors:** Bin Liu, Fang Yang, Tian Wen Zhang, Jiachang Tan, Zhenchao Yuan

**Affiliations:** ^1^ Department of Orthopaedic Soft Tissue Surgery, Guangxi Medical University Cancer Hospital, Nanning, ;China; ^2^ Department of Orthopaedic Medical Records Library, Guangxi Medical University Cancer Hospital, Nanning, ;China; ^3^ Guangxi Medical University Graduate School, Nanning, ;China

**Keywords:** 3D printed pelvic prosthesis, endoprosthetic failure, complications, clinical exploration, limb salvage

## Abstract

**Background:**

The use of 3D-printed pelvic prosthesis for postoperative reconstruction after pelvic tumor resection has become one of the primary reconstruction methods the incidence of complications related to postoperative prosthesis reconstruction is high. Drawing on the failure of the type of bone tumor reconstruction in Henderson,the occurrence of postoperative complications was explored to take advantage of the design improvement of the 3D-printed prosthesis of subsequent pelvic tumors.

**Methods:**

The data for patients who underwent 3D-printed pelvic tumor prostheses in the Department of Bone and Soft Tissue Surgery at the Affiliated Cancer Hospital of Guangxi Medical University from January 2019 to October 2022 were collected and analyzed.

**Results:**

The median follow-up time for all patients was 15.99 months (1.33-31.16 months). At the most recent follow-up,all patients were alive,with an average Musculoskeletal Tumor Society (MSTS) score of 21.46 (17 to 26 points). Local recurrence occurred in two cases (15.3%), metastasis in four cases (30.7%), and complications in 10 cases (76.9%). Early complications after surgery were primarily local wound fissure, deep tissue infection, and postoperative neuralgia. Later complications included loose dissolution of internal fixation, postoperative prosthetic dislocation, and postoperative gluteal middle muscle gait.

**Conclusion:**

3D printing personalized design pelvic tumor prosthesis is an effective way to reconstruct, and designing pelvic 3D printed tumor prosthesis with the help of Henderson’s bone tumor reconstruction failure concept may help bone tumor surgeons develop better pelvic tumor prosthesis.

## Background

Extensive surgical resection is the mainstay of treatment for primary bone tumors (1), and effective postoperative reconstruction using modular prostheses after pelvic tumor resection used infrequently compared to extremity bone tumors. Currently,the main alternatives to tumor prosthesis reconstruction include allograft pelvic reconstruction, autologous pelvic inactivation reconstruction, joint fusion, and joint replacement (2). Using these reconstruction methods may bring about adverse effects and complications. Based on the above status quo leads to an urgent clinical need for a stable and effective implant prosthesis with fewer complications. With the promotion and application of medical 3D printing technology, the clinical application of 3D-printed pelvic tumor prostheses has become possible in recent years. While the clinical application of 3D-printed pelvic tumor prostheses has brought many convenient reconstructions, the complications associated with the reconstruction have also presented new challenges to oncologic surgeons. This study aimed to collect clinical data related to postoperative pelvic reconstruction using 3D printed pelvic tumor prostheses from 13 patients from the Department of Bone and Soft Tissue Surgery of the Affiliated Cancer Hospital of Guangxi Medical University and to apply Henderson’s concept of failure for bone tumor reconstruction for relevant analysis, to effectively guide the design and clinical application of 3D-printed pelvic tumor prosthesis.

## Materials and methods

A retrospective analysis was conducted on all patients who underwent pelvic tumor resection and applied 3D printed pelvic tumor prosthesis reconstruction in the Department of Bone and Soft Tissue Surgery, Cancer Hospital,Guangxi Medical University,from January 2019 to October 2022.

Whole-body contrast-enhanced computerized tomography (CT), and locally enhanced magnetic resonance imaging (MRI) were performed in all cases to assess the whole-body condition and surgical margins of tumors. The Enneking stage system assessed tumor progression based on the tumor pathological grade, local invasion extent, and distant metastases. We collected data from patients voluntarily and entered into a verbal agreement, which was accepted and approved by the Institutional Review Board of the Cancer Hospital of Guangxi Medical University. We confirm that our study complies with the Declaration of Helsinki.

### 3D-printed custom pelvic prosthesis design and preparation

**Figure 1:**
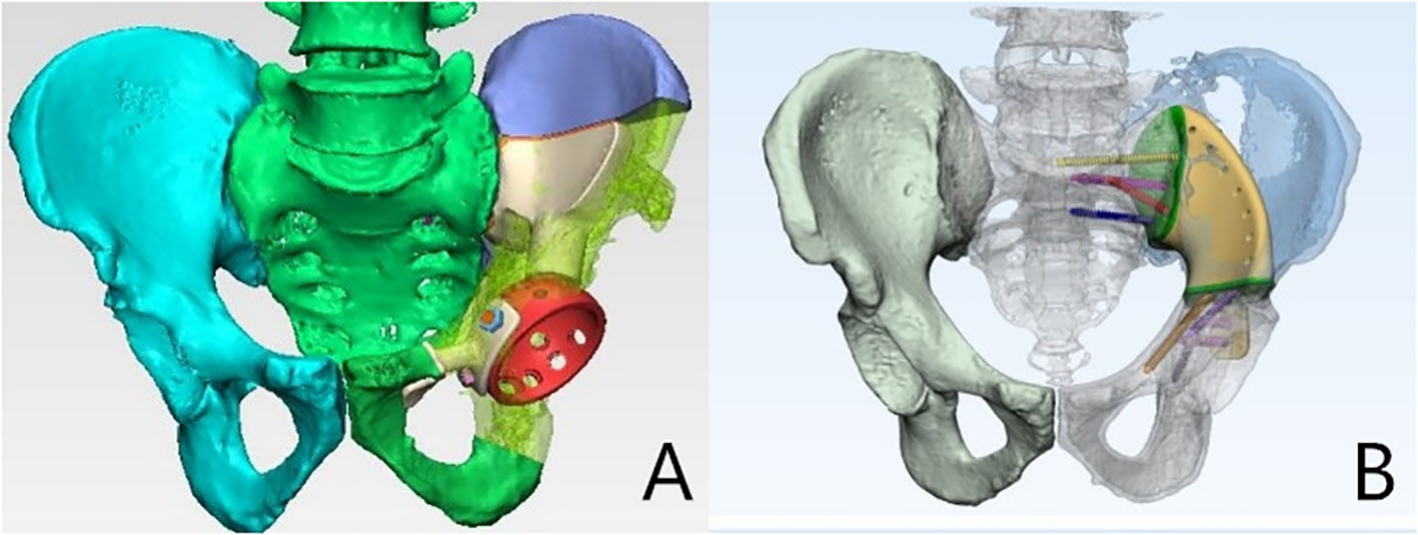
Pelvic tumour prosthesis design is personalised. **(A)** Pelvic tumour involving I+ II+ IV; **(B)** Pelvic tumour involving II+ IV.

Preoperative puncture pathology was performed in all cases. Two pathologists reviewed the results, and preoperative multidisciplinary discussions on treatment options were held to ensure the best possible treatment. Chemotherapy or radiotherapy is performed as needed. In addition, Dicom files from CT scans with a layer thickness of 0.625 mm were sent to the custom tumor prosthesis manufacturer to facilitate the 3D-printing of a computer-simulated preoperative reconstruction of the pelvic tumor prosthesis. All pelvic tumor prostheses were 3D-printed using Ti6A14V titanium alloy powder according to preoperative design; calcium hydroxyphosphate was used to coat the fusion site with normal bone. The surgical team also determined the extent of tumor invasion based on the MRI and the extent of surgical osteotomy by combining medical engineering to tailor the pelvic tumor prosthesis to the patient’s tumor condition. See **Figure 1**.

### Surgical procedures

The same operator performed all surgeries in this group. All patients were placed in a lateral floating position, and surgical resection of the pelvic tumor was performed according to the pelvic tumor, using a combined surgical incision with a partial Smith-Peterson (SP) approach,mainly in the anterior part (3). An enlarged Gibson approach was primarily used in the posterior part (4). Precise pelvic tumor resection was performed using 3D printed osteotomy guides, and assisted fixation was accomplished using a phase I of bone cement for pelvic prostheses using an internal spinal fixation system. Osteotomies were performed using the preoperative design to install the 3D-printed pelvic prosthesis. Soft tissue reconstruction was performed using muscles such as the gluteus maximus and external rotator muscle groups to surround the prosthesis as much as possible to reduce soft tissue gaps and reduce the risk of late infection. Postoperative intravenous antibiotics were routinely used to prevent infection until three days after extubation. Wound drains usually lasted for 5-10 days. Wound extubation was performed when the wound drainage was < 50 ml per day.

### Follow-up

**Figure 2:**
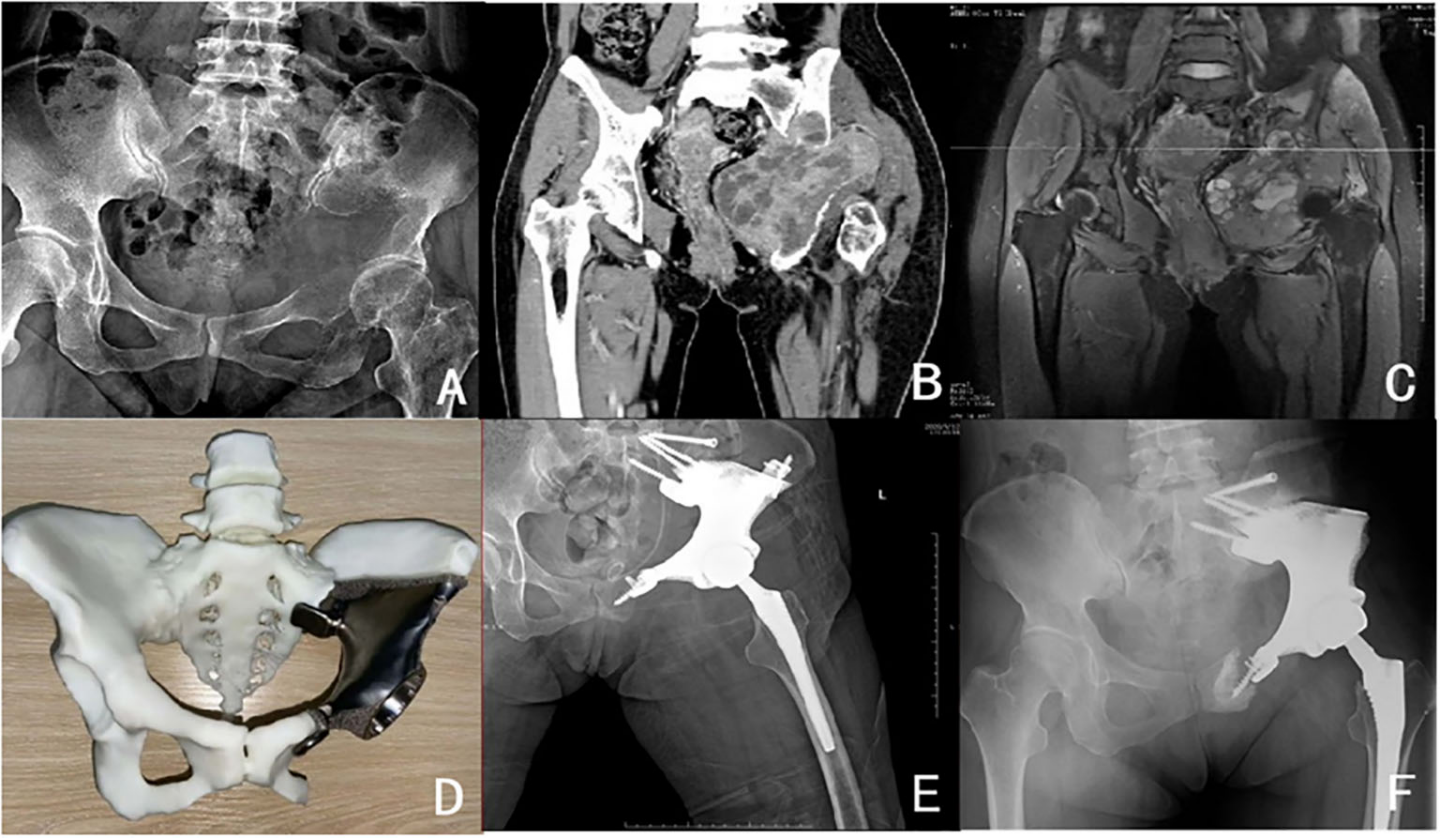
Patient,female,36 years old,pathology of malignant giant cell tumour of bone **(A)** Preoperative pelvic X-ray; **(B)** Preoperative pelvic CT; **(C)** Preoperative pelvic MRI; **(D)** Preoperative pelvic 3D printed tumour prosthesis; **(E)** Postoperative review pelvic X-ray; **(F)** Postoperative loosening of symphysis pubis fixation device.

All patients underwent postoperative radiographs and CT scans to check the positioning of the internal fixation of the prosthesis and screws every three months for the first two years, every four months for the third year, and every six months for the fourth and fifth years. The MSTS score reflected functional outcomes and patient satisfaction (5). It was used at the final follow-up to assess postoperative function, complications including wound dehiscence and infection, prosthesis dislocation and loosening, and oncological assessment, including local recurrence and metastasis. Postoperative wound dehiscence was treated by resuturing, and local infection was treated by infection debridement. All tumor prostheses were not removed. See **Table 1** and **Figure 2**.

**Table 1:** Clinical features associated with patients with pelvic tumours.

No.	Age	Gender	Pathological diagnosis	Stage	Lesion	Surgical margins	Early complications	Late complications	Follow-up (Month)	Local recurrence	Metastasis	MTST
1	32	Female	dedifferentiated chondrosarcoma	IIB	II+III	Marginal resection	Wound dehiscence	None	1.3	None	None	26
2	60	Female	Highly differentiated chondrosarcoma	IIB	II+III	Marginal resection	Wound dehiscence	Loose internal fixation	3.1	None	None	19
3	48	Male	Chondrosarcoma	IIB	I+II+IV	Marginal resection	Postoperative neuralgia	Postoperative neuralgia	2.5	None	None	22
4	16	Male	Giant cell tumour of bone	IIB	I+II+IV	Marginal resection	None	None	4.9	None	None	25
5	51	Male	Melanoma	III	II+III	Marginal resection	Postoperative neuralgia	Postoperative neuralgia	13.8	Yes	Yes	19
6	19	Male	Osteosarcoma	IIB	I+II+IV	Marginal resection	None	None	14.7	None	None	22
7	57	Female	Thyroid cancer	III	I+II	Marginal resection	Postoperative infection	Loose internal fixation	7.5	Yes	Yes	21
8	16	Female	Ewing sarcoma	IIB	I+II+IV	Extended resection	Gluteus medius gait	Gluteus medius gait	26	None	None	17
9	66	Female	Lung adenocarcinoma	III	II	Extended resection	None	None	13.6	None	Yes	21
10	22	Male	Ewing sarcoma	IIB	I+II+IV	marginal resection	None	Postoperative dislocation	22.4	None	None	25
11	41	Male	Chondrosarcoma	IIB	II	marginal resection	None	Loose internal fixation	21.8	None	None	22
12	36	Female	Malignant giant cell tumour of bone	III	II+IV	marginal resection	Postoperative infection	Loose internal fixation	31.2	None	Yes	19
13	43	Male	Chondrosarcoma	IIB	I+II+IV	marginal resection	None	None	3.8	None	None	21

ISOLS, International Society of Limb Salvage; MSTS, Musculoskeletal Tumor Society; CT, Computed tomography; MRI, Magnetic resonance imaging.

## Results

There were 13 patients with pelvic tumors, including seven males and six females, with a mean age of 32. All cases underwent C-arm guided local bone puncture-biopsy. There were seven histological types: chondrosarcoma (5 cases), Ewing sarcoma (2 cases), malignant giant cell tumor of bone (2 cases), osteosarcoma (1 case), thyroid metastases (1 case), metastatic lung adenocarcinoma (1 case), and metastatic melanoma (1 case). Regarding the Enneking stage,there were nine cases in Stage IIB and four in Stage III. The pelvic tumor sites were classified according to the Dunham classification. The tumors in Region I were limited to the iliac wing, Region II to the pelvic acetabulum, Region III to the obturator region composed of the pubic stop and the sciatic branch, and Region IV to the sacrum and sacroiliac joint (6). The median follow-up time was 15.99 months (1.33-31.16 months). A ll patients survived during the most recent follow-up with a mean MSTS score of 21.46 (range 17-26). Local recurrence occurred in 2 cases (15.3%), metastases in 4 cases (30%),complications occurred in 10 cases (76%), early postoperative complications were mainly local wound dehiscence (2/13), deep tissue infection (3/13), postoperative neuralgia (2/13), late complications were mainly loosening of internal fixation (4/13),postoperative prosthesis dislocation (1/13), and gluteus medius gait (1/13).

## Discussion

For patients with pelvic tumors,extensive surgical resection is a standard method for surgical removal of pelvic tumors with limited coverage due to extensive removal of pelvic soft tissues. Reconstruction of bone defects after tumor removal using traditional custom-made pelvic tumor prosthesis implants is an effective but high-complication procedure. In recent years, the application of 3D printing technology for pelvic tumor prosthesis reconstruction can help to restore the function of patients’ lower limbs, especially in pelvic tumor cases facing massive bone defects that are difficult to recover. Applying 3D-printed custom-made pelvic prostheses can effectively improve the preservation of limb function. At the same time, the clinical practice of relevant cases can reduce the occurrence of 3D-printed pelvic prosthesis failure and optimize the custom-made pelvic tumor prosthesis to restore the lower limb. The clinical practice of 3D-printed pelvic prostheses can also reduce the incidence of 3D-printed pelvic prosthesis failure and optimize the restoration of lower limb function with customized pelvic tumor prostheses. Following pelvic tumor resection, 3D printed pelvic tumor prostheses have enabled timely and effective postoperative stabilization and reconstruction of patients, while accompanying complications in the medium to long-term postoperative follow-up is more common. A complication rate of 76% was determined in this study. Local postoperative wound dehiscence,deep tissue infection,and postoperative neuralgia were the early complications, while the later complications were internal fixation loosening,postoperative prosthesis dislocation, and gluteus medius gait. Considering the high incidence of postoperative prosthesis failure and potential postoperative-related complications, future complications may need to be considered at the beginning of the design of the oncologically personalized 3D-printed pelvic tumor prosthesis.

In 2014, Henderson et al. (3) classified prosthetic failures requiring revision surgery into five subtypes: soft tissue failure,aseptic loosening, structural failure, infection, and local tumor progression or recurrence. Based on these subtypes, these factors are considered in the design of the pelvic prosthesis. After soft tissue reconstruction, tumors in the acetabular pelvis often accumulate lesions in the iliopsoas and gluteus medius muscles, and extensive resection of the pelvic muscle groups often results in a lack of muscle power. It is possible to design relevant muscle attachment points for tumor prostheses to maximize the retention of relevant power muscle stops under conditions of complete tumor resection.

Aseptic loosening: Increased stress on the prosthesis may lead to loosening of the prosthetic contact surface due to extensive soft tissue deficiencies resulting from extended resection. The use of biological prosthetic reconstruction can effectively improve the occurrence of such loosening. The optimal design of the sacroiliac joint using the pelvic 3D-printed microporous design structure facilitated the growth of the fused bone into the permanently fused sacroiliac joint, with the aid of preoperatively designed screws to enhance fixation and prevent early loosening of the prosthesis. The optimal solution was further analyzed with finite element analysis of the fixation device (7). Pelvic ring repair can be a strategy with or without pelvic ring reconstruction, depending on tumor resection. Most authors agree on the importance of restoring the connection between the sacroiliac joint, acetabulum, and pubic symphysis (8),while a few support non-anatomical reconstruction (9).

Structural failure: Hip reconstruction uses an oncoplastic prosthetic cup fixed within the prosthetic acetabulum, with a high-sided PVC acetabular cup to reduce the risk of femoral prosthesis dislocation; in addition, the double-acting head acetabular design transfers lower limb stresses transmitted by the restrained hip prosthesis directly to the bone-prosthesis interface,effectively reducing the risk of prosthesis failure because of better cushioning. The combined application of these two strategies effectively prevents dislocation of the femoral head.

Infection: Postoperative infection is associated with a sizable postoperative defect after tumor resection. Using locally coated silver pelvic prostheses reduces the risk of postoperative prosthetic infection by reducing the creation of postoperative residual cavities. In contrast,the design of the glossy surface of the tumor prosthesis may reduce the chance of infection. Using an antibacterial hydrogel coating on the surface of the prosthesis may reduce biofilm formation. These initiatives effectively reduce infection and antibiotic resistance (10). In addition, 3D-printed pelvic prostheses attempt to achieve a 1:1 reconstruction of the normal pelvic anatomy. This can lead to high postoperative incisional tension, local skin necrosis and wound dehiscence, which can be alleviated using equivalently scaled or simplified prostheses. For example, in the case of tumors involving the iliac wing, the tumor prosthesis can be attached directly to the sacroiliac joint to simplify the shape of the iliac wing.

This study reported two cases (15.3%) of local tumor recurrence. The survival rate of patients after pelvic reconstruction needs to be cautiously assessed regarding the size of the tumor, the extent of invasion, and the pathological specificity. Studies have reported a five-year survival rate of 55% (11). Combined with these data, pelvic tumor prosthetic reconstruction may benefit younger patients or patients with pathology suggestive of hyper differentiation. Younger patients are more likely to tolerate the procedure and adapt to function after surgery. Extensive surgical resection may require consideration of the choice between a custom-made prosthesis and an adjustable modular prosthesis to reduce tumor recurrence, leading to failure of the tumor prosthesis while balancing postoperative prosthetic function. Obtaining good function postoperatively while preserving limb function is particularly important for more invasive and complicating pelvic tumor surgery. The results of this study showed that patients achieved relatively effective function postoperatively,with a mean MSTS score of 21.46 (range 17-26) at the last follow-up visit. Furthermore, in a retrospective case study,Abudu et al. reported a mean MSTS score of 21 in 35 patients (12). These results emphasize the need for structural stability reconstruction of the pelvis. Thus, 3D-printed pelvic prostheses can reconstruct the stability of the pelvic structures in a timely and effective manner, allowing for good function in the postoperative reconstruction of patients with pelvic tumors.

Several limitations exist in this study. First, because the retrospective design and associated questions were primarily from our study center, the individualized design of the tumor patient data and associated clinical design data may lack generalizability. Second, the low prevalence of pelvic tumor cases,the small follow-up sample size, the absence of a study control group, and the scarcity of medium to long-term postoperative follow-up data following surgical resection and reconstruction are all inevitably present.

The risk of complications after 3D-printed tumor prosthesis for pelvic tumor resection and reconstruction are high. Designing pelvic 3D-printed tumor prostheses guided by the Henderson concept of failed staging for bone tumor reconstruction is an effective attempt to achieve an individualized design of appropriate tumor prostheses for pelvic tumor patients,maximizing the patient’s higher pelvic stability and postoperative limb function. This approach may help the bone oncology surgeon to develop a better concept for pelvic tumor design, which could be used for more eligible patients. Finally, with advances in material science and 3D-printing technology, a new generation of 3D-printed custom pelvic implants will be evaluated under this concept to maximize their effectiveness in clinical practice.

## Data availability statement

The raw data supporting the conclusions of this article will be made available by the authors, without undue reservation.

## Ethics statement

The studies involving humans were approved by the Institutional Review Board of the Cancer Hospital of Guangxi Medical University. The studies were conducted in accordance with the local legislation and institutional requirements. Written informed consent for participation was not required from the participants or the participants’ legal guardians/next of kin in accordance with the national legislation and institutional requirements. The manuscript presents research on animals that do not require ethical approval for their study. Written informed consent was obtained from the individual(s) for the publication of any potentially identifiable images or data included in this article.

## Author contributions

BL: Writing – original draft, Writing – review & editing. FY: Writing – original draft, Writing – review & editing. TZ: Writing – original draft, Writing – review & editing. JT: Validation, Visualization, Writing – original draft, Writing – review & editing. ZY: Writing – original draft, Writing – review & editing.
